# Diagnostic performance of an ultra-sensitive RDT and a conventional RDT in malaria mass testing, treatment and tracking interventions in southern Ghana

**DOI:** 10.1186/s13071-024-06354-x

**Published:** 2024-07-01

**Authors:** Linda Eva Amoah, Ndong Ignatius Cheng, Festus Kojo Acquah, Susan Adu-Amankwah, Dorcas Gyama Bredu, Benedicta A. Mensah, Sherik-fa Anang, Bernice Cubson Abban, Abena Busayomi, Sebastian Shine Kwarpong, Prosper Kofi Tey, Elizabeth Cudjoe, Alexander Asamoah, Tobias McKenzie Holden, Jaline Gerardin, Justice Nonvignon, Collins Ahorlu

**Affiliations:** 1 Department of Immunology, Noguchi Memorial Institute for Medical Research, University of Ghana, Accra, Ghana; 2 Department of Epidemiology, Noguchi Memorial Institute for Medical Research, University of Ghana, Accra, Ghana; 3 National Malaria Control Program, Accra, Ghana; 4 Department of Preventive Medicine and Institute for Global Health, Northwestern University Feinberg School of Medicine, Chicago, IL USA; 5 Department of Health Policy, Planning and Management, School of Public Health, College of Health Sciences, University of Ghana, P. O. Box LG13, Legon, Ghana

**Keywords:** Malaria, RDT, Ultra-sensitive, MTTT, Ghana, PET-PCR

## Abstract

**Background:**

Application of numerous malaria control interventions has led to reduction in clinical malaria cases and deaths but also the realisation that asymptomatic parasite carriers play a key role in sustaining transmission. This study assessed the effectiveness of using the Ultra-sensitive NxTek eliminate RDT (uRDT) and conventional SD Bioline HRP2 RDT (cRDT) in diagnosing asymptomatic parasitaemia while measuring the impact of mass testing, treatment and tracking (MTTT) on the prevalence of asymptomatic malaria over a 1-year period in Ghana.

**Methods:**

A total of 4000 targeted participants from two towns, Obom and Kofi Kwei, with their surrounding villages, were tested for asymptomatic malaria four times over the study period using uRDT (intervention) and the cRDT (control) respectively. Participants carrying malaria parasites were followed by home visit and phone calls for compliance to treatment, and filter paper blood blots collected from participants were used to determine true parasite carriage by PET-PCR. A mathematical model of the study site was developed and used to test the impact of test sensitivity and mass migration on the effect of MTTT.

**Results:**

The start and end point sensitivities of the cRDT were 48.8% and 41.7% and those for the uRDT were 52.9% and 59.9% respectively. After a year of MTTTs, asymptomatic parasite prevalence, as determined by PCR, did not differ statistically in the control site (40.6% to 40.1%, *P* = 0.730) but decreased at the intervention site (55.9% to 46.4%, *P* < 0.0001). Parasite prevalence by RDT, however, indicated statistical reduction in the control site (25.3% to 22.3%, *P* = 0.017) and no change in the intervention site (35.1% to 36.0%, *P* = 0.614). The model predicted a mild effect of both diagnostic sensitivity and human movement in diminishing the impact of MTTT in the study sites.

**Conclusions:**

Asymptomatic parasite prevalence at the molecular level reduced significantly in the site where the uRDT was used but not where the cRDT was used. Overall, the uRDT exhibited higher sensitivity relative to the cRDT. Highly sensitive molecular techniques such as PET-PCR should be included in parasite prevalence estimation during MTTT exercises.

**Graphical Abstract:**

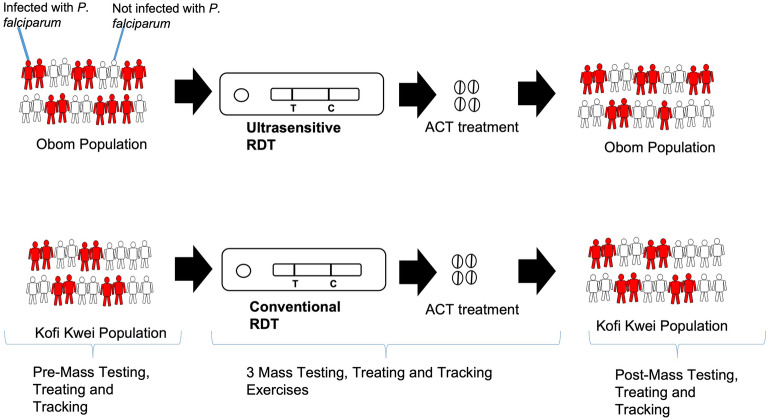

**Supplementary Information:**

The online version contains supplementary material available at 10.1186/s13071-024-06354-x.

## Background

Over the past decades several interventions have been made to control and eliminate malaria in endemic countries. These include distribution of insecticide-treated nets (ITNs), intermittent preventive treatment of malaria in infants (IPTi) and in pregnant women (IPTp), indoor residual spraying (IRS) and seasonal malaria chemoprevention (SMC) programmes [1, 2]. With the availability of Rapid Diagnostic Tests (RDTs) for point-of-care diagnosis, prompt diagnosis and early treatment of malaria with artemisinin-based combination therapy has contributed to control and elimination efforts against the disease [3].

However, asymptomatic infections continue to pose a threat to malaria elimination programmes, as asymptomatic parasite carriers present no clinical symptoms and thus are untreated. Persistent asymptomatic parasite carriage has even been linked with a lower likelihood of developing symptomatic malaria [4, 5]. In most malaria-endemic settings, asymptomatic infections are reported to be more common than symptomatic ones [6, 7], serving as reservoirs for transmission [8–10]. In the past few years, mass drug administration (MDA) has been employed to target asymptomatic parasite carriage in a population to reduce transmission [11, 12]. Though this approach is conceded with challenges such as coverage, cost, refusal to take the drug (as most people who do not present clinical symptoms think treatment is needless [13]) and drug pressure on individuals with the parasite, which subsequently may lead to parasite resistance [11], many studies have reported a decline in malaria cases owing to the intervention of MDA [12, 14].

Thus, similar intervention approaches such as mass testing, treatment and tracking (MTTT) are of interest as alternatives for MDA [15]. Here, community household screening is done to identify individuals with the malaria parasite and treat them with an anti-malarial drug without necessarily administering drugs to the entire population as is done in the case of MDA. The reduction in drug administration, prevention of drug pressure and development of resistance are the major advantages of MTTT. Also, in MTTT individuals with malaria treatment are followed up to ensure adherence to treatment. A recent study in Ghana has demonstrated that MTTT can contribute to the reduction of asymptomatic infection as well as symptomatic malaria cases in endemic communities [15].

The limited sensitivity of RDTs may hinder the success of MTTT for elimination as low parasite densities (< 200 parasite/µl) may be missed [3]. Thus, highly sensitive point-of-care tools for detection of low parasite densities may be required for an effective MTTT intervention. Recent development of new RDTs such as the novel ultra-sensitive Alere™ Malaria Ag *Plasmodium falciparum* RDT (uRDT) can detect parasite densities as low as 10–40 p/µl compared to 100–200 p/µl detection by current available RDTs [16]. A work by Landier et al. reported a sensitivity of field testing of asymptomatic parasitaemia of 34.8% and 15.2% for uRDT and standard RDT respectively [17], while a previous pilot study reported sensitivity of uRDT and standard RDT to be 73.0% and 58.0% respectively. Although the uRDT does not have the same sensitivity as molecular tools, its ability to detect additional parasitaemia lower than the detection limits of standard RDTs makes it a better choice than conventional RDTs for field surveys. In addition to the novel ultra-sensitive Alere™ Malaria Ag *Plasmodium falciparum* RDT, the NxTEK Eliminate RDT has also been shown to detect about 10% more parasitaemia than the standard RDTs [18, 19]. However, more studies assessing the performance of uRDTs in community-scale interventions such as MTTTs need to be carried out to provide more basis for their use or otherwise in such interventions. This study assessed the diagnostic properties of the NxTEK Eliminate RDT against the standard RDTs (SD Bioline) using a highly sensitive molecular tool, PET-PCR, as a reference tool and the impact of their utilization in a year of MTTT. Mathematical modelling was used to assess the impact of test sensitivity and human movement in the study area on the outcome of MTTT.

## Methods

### Study site and design

The study was a prospective study that utilized convenience sampling in two main towns, Obom and Kofi Kwei, and their surrounding communities, randomly assigned as the intervention and control arms respectively. Both sites are located in a high malaria transmission setting of the Ga south municipality of the Greater Accra Region of Ghana. The two communities are separated by about 5 km with residents from both communities living in compounds comprising of different subfamilies who mainly rely on subsistence farming. Each community is comprised of a small cluster of settlements, with those in Kofi Kwei mostly named after families occupying that setting whereas the settings in Obom are mainly named based on landmarks. In the Obom community, seven settings including Palace, Chief Imam, Carpenter, Lovekope, Quarters, Assemblies and Lebene were selected for this study. In the Kofi Kwei community, five settings including Pabiman, Ayitey Kortor, Kobla Odeba, Kwayi and Mamomo were selected.

The study used a 13-month mass testing, treatment and tracking (MTTT) exercise comprising quarterly MTTT implementation exercises conducted in September 2020, January, May and September 2021 combined with home-based management of symptomatic cases during the periods in between the MTTT surveys. The NxTEK Eliminate RDT kit (05FK140) was used at Obom, the intervention site, whilst the SD Bioline malaria RDT (05FK50-40-0) was used in Kofi Kwei, the control site.

### Training and implementation of MTTT

To ensure maximum participation, community-based health volunteers (CBHVs) and nurses were trained at the beginning of the study and provided with monthly refresher training sessions. Following the training, CBHVs conducted a census in both communities, registered all households and assigned a unique ID to every member of each household.

For each round of MTTT, individuals from every household were tested with either the cRDT (control arm) or uRDT (intervention arm). Individuals with a positive RDT result were treated with a first-line antimalarial, artemether-lumefantrine, an artemisinin-based combination therapy (ACT), except for special cases such as pregnant women and infants who were referred to the clinic for treatment. During all four intervention rounds, participants who tested positive for malaria were tracked by a follow-up visit at their homes at least once to ensure compliance to the treatment regimen. In addition, contacts of the parasite-positive individuals or their household heads were obtained for a subsequent follow-up call for enquiry and advice for compliance and completion of medication. In addition to on-site diagnosis and treatment, the trained volunteers provided home-based treatment for all participants who became symptomatic of malaria and tested positive at any point in time. Participants were considered missed if they had moved out or relocated out of the community or were visited at least three times and were unreachable. During each visit, individuals who had moved into the community or were not recruited prior to the first MTTT and were willing to partake of the study were invited to provide informed written consents and recruited into the study.

### Sample collection during MTTT

During each MTTT, asymptomatic malaria screening by researchers with the help of the nurses and CBHVs was accomplished by visiting the residents in their homes. Following completion of written informed consent, demographic features such as age, sex and bednet use were obtained. The axillary temperature of each participant was captured using a digital hand-held thermometer. About 150 μl finger-pricked blood was obtained from each participant to spot a Whatman no. 3 filter paper as well as an RDT (NxTEK Ultra-sensitive or SD Bioline HRP2 RDT) following the manufacturer’s instruction. The blood-stained Whatman no. 3 filter papers were air dried and each dry blood spot (DBS) was kept in individual Ziploc bags containing a desiccant and stored at 4 °C until processed (beginning a week after each collection). In addition, 5 μl finger-pricked blood of all children < 15 years old was used to determine haemoglobin levels using the Urit 12 haemoglobin metre.

### Sample collection after MTTT

Home-based testing was implemented during the periods between two successive MTTTs. Any febrile study participant contacted a CBHV for a malaria RDT (NxTEK Ultra-sensitive or SD Bioline HRP2 RDT) test, with DBS preparation as described above and treatment with artemether-lumefantrine.

### Laboratory analysis

#### DNA extraction

Genomic DNA isolation from the DBS was carried out using the Chelex method [20]. Briefly, two 3-mm-diameter discs were punched out of the DBS into a sterile 1.5-ml microfuge tube. An aliquot of 1 ml of 0.5% Tween-phosphate-buffer saline (PBST) was added to the tube, vortexed and incubated with shaking overnight at room temperature. The tubes were subsequently centrifuged at 12,300×*g* for 2 min and the supernatant aspirated. Next, 1 ml of PBS was added, vortexed and incubated at 4 °C for 30 min. The supernatant was aspirated; lastly, 100 μl of nuclease-free water and 50 μl of 20% Chelex were added to the tube, incubated at 95 °C for 10 min with intermittent vortexing every 2 min. The tubes were finally centrifuged at 12,300×*g* for 8 min and the supernatant was transferred into a sterile 0.5-ml microfuge tube and used immediately or stored at − 20 °C.

#### Parasite carriage determination by PET-PCR

PET-PCR assay as previously described by Lucchi et al. [21] was performed for all samples, with slight modification as described by [22]. Briefly, multiplex amplification of the 18s rRNA gene (*Plasmodium* spp.) and r364 gene (*P. falciparum*) was performed in a 15-μl reaction containing 2X TaqMan Environmental Master Mix 2.0 (Applied BioSystems) and 0.25 μM each of forward and reverse primers except for the *P. falciparum* HEX-labelled primer whose concentration was 0.125 μM and 2 μl of DNA template. A tenfold serial dilution of NF54 wild-type *P. falciparum* DNA with an initial concentration of 12.32 ng/μl was used to generate a standard curve to quantify *P. falciparum* present in each sample. The reactions were performed under the following cycling parameters: initial hot-start at 95 °C for 15 min, followed by 45 cycles of denaturation at 95 °C for 20 s, annealing at 63 °C for 40 s and extension at 72 °C for 30 s. All assays were performed using the QuantStudio™ 3 and 7500 Fast thermocyclers (Applied BioSystems).

The correct fluorescence channel was selected for each fluorescently labelled primer set and the cycle threshold (CT) values recorded at the end of the annealing step. An in-house laboratory determined limit of detection for the CT of 41.819 for assays in which the negative controls read ‘undetermined’ were used as cut off for determining positivity. For assays in which the negative control gave a value, a sample is assigned positive if it has a CT value lower than that of the negative control. The parasite density in each sample was also extrapolated from a standard curve of CT values for standard samples of known *P. falciparum* parasite density.

#### Mathematical modelling

The individual-based mathematical model EMOD v2.20 [23] was used to simulate malaria transmission, human migration, treatment-seeking and study interventions. EMOD includes a climate-dependent vector life cycle, explicit parasite densities, acquired immunity and pharmacokinetics and pharmacodynamics of antimalarial drug effects [24–27]. A spatial model was constructed at the village level, including the two study villages and two generic villages representing areas outside the study area (Additional file; Table S3). Modelled malaria seasonality was set to reproduce the shape of monthly uncomplicated malaria cases observed at health facilities in the study site (Additional file; Figure S1) from September 2019 to November 2021 (Additional file; Figure S2). Baseline transmission intensity in the two study villages was calibrated by sampling the availability of larval habitats and case management rates in a full grid search, then calculating the Euclidian distance between modeled and observed PCR parasite prevalence in September 2020 (Table 2) (Additional file; Figure S3). The 10 parameter sets with the shortest distance were used in subsequent simulations.

MTTT interventions were modeled by testing simulated individuals with an RDT followed by treatment with artemether-lumefantrine of all individuals who tested positive. Coverage of MTTT was assumed to be 100% of at-home residents—all individuals were screened in the simulation unless they were away from the study villages. These assumptions of perfect screening coverage and adherence to treatment are reflective of the trial’s thorough follow-up for detected infections. Ultrasensitive (Obom) and conventional (Kofi Kwei) RDTs were modelled with sensitivities and specificities (Additional file; Figure S4) based on their performance relative to PCR (Table 5), assuming a detection threshold of 0.1 parasites per microlitre for PCR.

All scenarios included baseline migration, where individuals moved between the study villages at a rate of five trips per year of 5 days’ duration each and between study and generic villages at a rate of one trip per year of 30 days’ duration. In scenarios with mass migration, individuals were also assigned migration patterns with probabilities based on the observed recruitment and retention of study participants (Additional file; Table S1): people recruited after the first MTTT round were assumed to originate from the generic villages, and individuals migrated to the generic villages during subsequent missed MTTT rounds. Trip start dates were uniformly distributed between 2 and 16 weeks after each MTTT round so that no mass migration occurred during the intervention. Trip durations were set to align with the schedule of visits and reproduce the observed inflows and outflows of people (Additional file; Figure S5).

Four model scenarios were simulated: (1) including distribution of MTTT, (2) including mass migration, (3) including both MTTT and mass migration and (4) no MTTT or mass migration. Two additional scenarios of MTTT were simulated, with and without mass migration, using a “perfect” RDT with modelled sensitivity of 1.0 and specificity of 1.0 relative to PCR.

Mathematical modelling methods are described in more detail in section III of the additional file.

## Results

**Fig. 1:**
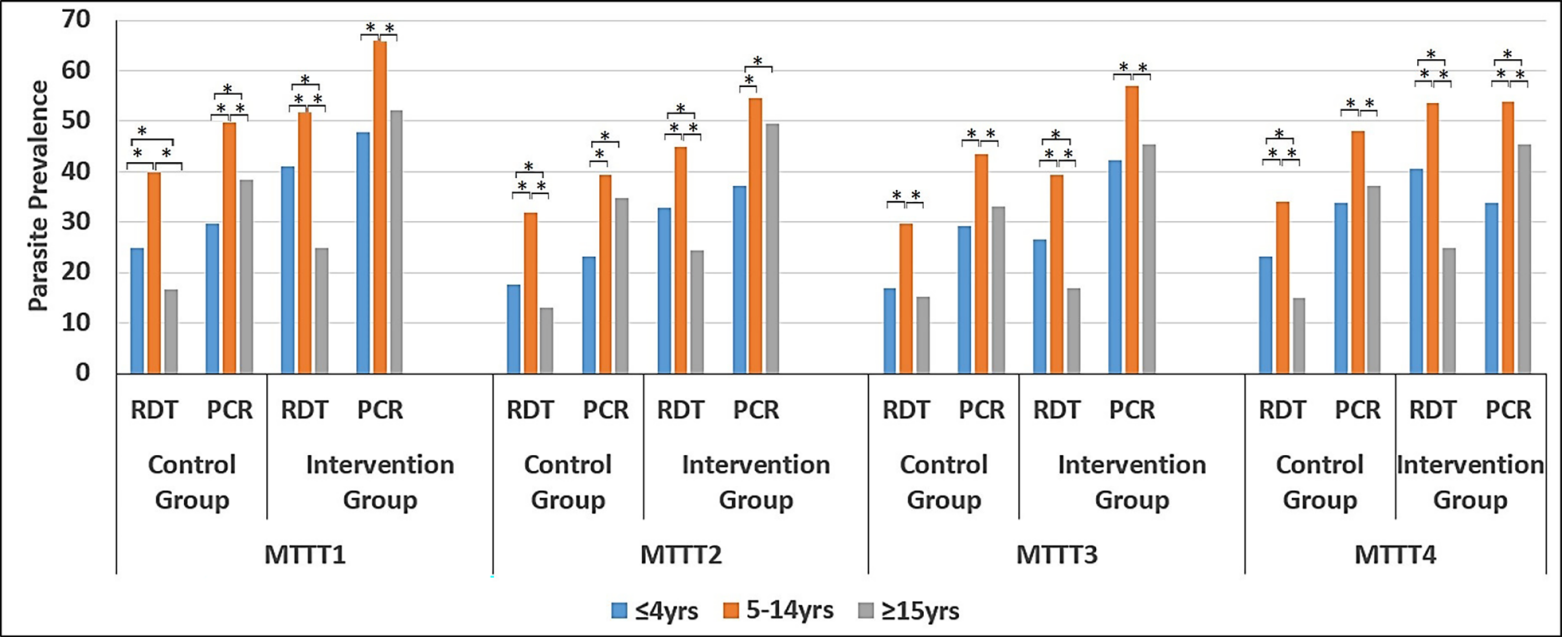
PCR and RDT prevalence of asymptomatic malaria with various age groups between the two arms of the study across the study period. *P-value < 0.05

The study successfully conducted four cross-sectional MTTTs for both the intervention arm and the control arm in September 2020, November 2020, March 2020 and September 2021. Children under < 5 years fewest while participants ≥ 15 years were the most abundant in both study sites during all visits (Table 1). Though the target number of participants was 4000 (1500 from the intervention arm and 2500 from the control arm), a total of 1438 (95.9%) participants were screened at baseline for the intervention arm and 2274 (90.0%) for the control arm. Including new participants, a total of 1441 (96.1%), 1426 (95.1%) and 1513 (100.9%) at the intervention arm and 2165 (86.6%), 2256 (90.2%) and 2388 (90.2%) at the control arm were screened during the second, third and fourth MTTTs respectively. In the intervention group, a total of 449 participants were present throughout all 4 MTTT while 844 were present throughout for the control group (Additional file; Table S1). Asymptomatic parasite carriage was generally much higher in the intervention arm than in the control arm (Table 1 and Fig. 1) in all four visits as determined by both RDT and PCR (Table 1).

**Table 1:** Demographics of study populations

Characteristics	MTTT1	MTTT2	MTTT3	MTTT4
Kofi Kwei (control group)				
Female gender, *n*/*N* (%)	1295/2243 (57.7)	1273/2165 (58.8)	1345/2255 (59.6)	1414/2386 (59.3)
Age group				
< 5 years	363/2243 (16.2)	361/2165 (16.7)	394/2255 (17.5)	404/2386 (16.9)
Aged 5 to 14 years	712/2243 (31.7)	715/2165 (33.0)	717/2255 (31.8)	752/2386 (31.5)
≥ 15 years	1168/2243 (52.1)	1089/2165 (50.3)	1144/2255 (50.7)	1229/2386 (51.5)
ITN use, *n*/*N* (%)	775/2234 (34.7)	749/2164 (34.6)	774/2255 (50.3)	779/2379 (32.7)
Malaria RDT, *n*/*N* (%)	568/2243 (25.3)	433/2165 (20.0)	453/2255 (20.1)	533/2386 (22.3)
Malaria PCR, *n*/*N* (%)	884/2178 (40.6)	731/2131 (34.3)	787/2208 (35.6)	954/2380 (40.1)
Obom (intervention group)				
Female gender, *n*/*N* (%)	834/1398 (59.7)	844/1428 (59.1)	843/1422 (59.3)	894/1508 (59.3)
Age group				
< 5 years	183/1398 (13.1)	195/1428 (13.7)	203/1422 (14.3)	231/1508 (15.3)
Aged 5 to 14 years	411/1398 (29.4)	463/1428 (32.4)	470/1422 (33.1)	461/1508 (30.6)
≥ 15 years	804/1398 (57.5)	770/1428 (53.9)	749/1422 (52.7)	816/1508 (54.1)
ITN use, *n*/*N* (%)	462/1393 (33.2)	576/1428 (40.3)	522/1412 (37.0)	436/1510 (29.0)
Malaria RDT, *n*/*N* (%)	491/1398 (35.1)	460/1428 (32.2)	366/1422 (25.7)	541/1502 (36.0)
Malaria PCR, *n*/*N* (%)	758/1357 (55.9)	698/1413 (49.4)	679/1394 (48.7)	693/1495 (46.4)

Three rounds of MTTT exercises were carried out over the course of 1 year. The end point assessment was conducted in the form of a fourth MTTT exercise. Parasite carriage determined by PCR showed that the intervention arm had a significant reduction in parasite carriage from 55.9 to 46.4% (*P* < 0.0001) while the control arm retained the same level of parasite prevalence of about 40% (Table 2). Conversely, assessment of parasite carriage by RDT (NxTEK Ultra-sensitive in the Intervention arm and SD Bioline HRP2 RDT in the control arm) used indicated that whereas the control arm had a significant reduction in parasite carriage from 25.3 to 22.3% (*P* = 0.017), the intervention arm did not have any statistically significant change in its parasite prevalence (Table 2).

**Table 2:** Prevalence of *Plasmodium falciparum* within study communities by RDT and PCR

	MTTT1	MTTT2	MTTT3	MTTT4	*P*-value (MTTT1 vs MTTT4)
RDT					
Control group	568/2243 (25.3)	433/2165 (20.0)	453/2255 (20.1)	533/2386 (22.3)	**0.017**
Intervention group	491/1398 (35.1)	460/1428 (32.2)	366/1422 (25.7)	541/1502 (36.0)	0.614
PCR					
Control group	884/2178 (40.6)	731/2131 (34.3)	787/2205 (35.7)	954/2380 (40.1)	0.73
Intervention group	758/1357 (55.9)	698/1413 (49.4)	679/1394 (48.7)	693/1495 (46.4)	**< 0.0001**

The reduced number of total PCR tests for each visit was due to missing DBS samples

*P* values of less than 0.05 are significant and are in bold

Children aged 5 to 14 years recorded a higher parasite prevalence than children < 5 years as well as participants ≥ 15 years. This trend was observed for parasite carriage estimated by both PCR and RDT and replicated in all MTTTs (Fig. 1).

Univariate analyses of the impact of the MTTT within specific groups of participants indicated that within the control group, parasite prevalence by RDT (cRDT) decreased in only children aged 5 to 14 years but not in participants < 5 years old or > 14 years. Furthermore, the decrease in parasite carriage was observed in males but not in females. Parasite prevalence did not change in the control group for both users and nonusers of mosquito net by RDT (Table 3). In the intervention group, age group, gender and bed net usage did not affect parasite carriage as determined by the uRDT.

**Table 3:** Univariate analysis of effect of MTTT interventions on RDT-determined prevalence of asymptomatic malaria over the study period

Characteristics	Control (Kofi Kwei)				Intervention (Obom)			
	MTTT1, *n*/*N* (%)	MTTT4, *n*/*N* (%)	*χ*^2^, *df*	*P* value	MTTT1, *n*/*N* (%)	MTTT4, *n*/*N* (%)	*χ*^2^, *df*	*P* value
Overall community	568/2243 (25.3)	533/2386 (22.3)	0.2542, 1	**0.017**	491/1398 (35.1)	541/1502 (36.0)	0.2542, 1	0.6141
Age group (years)								
< 5 years	902/363 (24.8)	94/404 (23.7)	0.2442, 1	0.6212	75/183 (41.0)	93/231 (40.3)	0.0022, 1	0.8816
Aged 5 to 14 years	284/712 (39.9)	256/753 (34.0)	5.456, 1	**0.0195**	216/411 (52.6)	246/459 (53.6)	0.094, 1	0.7589
≥ 15 years	194/1168 (16.6)	183/1229 (14.9)	1.336, 1	0.2478	200/804 (24.9)	202/8012 (24.9)	3 × 10^–7^, 1	0.9995
Gender								
Male	298/948 (31.4)	255/972 (26.3)	6.328, 1	**0.0119**	231/564 (41.0)	257/612 (42.0)	0.1298, 1	0.7187
Female	270/1295 (20.9)	278/1414 (19.7)	0.5921, 1	0.4416	260/834 (31.2)	284/890 (31.9)	0.1077, 1	0.7428
Bed net use								
No	193/775 (24.9)	191/779 (24.5)	0.0308, 1	0.8605	342/931 (36.7)	381/1063 (52.7)	0.1711, 1	0.4137
Yes	293/756 (38.8)	315/776 (40.6)	0.5394, 1	0.4627	147/462 (31.8)	158/436 (36.2)	1.954, 1	0.1621

*N* total number tested, *n* number that tested positive by RDT

*P* values of less than 0.05 are significant and are in bold

Univariate analyses using PCR-determined parasite carriage showed no difference in the parasite carriage of participants from the different age groups, gender or bednet use status in the control arm. In the intervention arm, however, a significant reduction of parasite carriage was observed in all the different categories in which participants were grouped based on the age, gender or usage of bed nets (Table 4).

**Table 4:** Univariate analysis of effect of MTTT interventions on PCR-determined prevalence of asymptomatic malaria over the study period

Characteristics	Control (Kofi Kwei)				Intervention (Obom)			
	MTTT1, *n*/*N* (%)	MTTT4, *n*/*N* (%)	*χ*^2^	*P* value	MTTT1, *n*/*N* (%)	MTTT4, *n*/*N* (%)	*χ*^2^	*P* value
Overall community	884/2178 (40.6)	954/2380 (40.1)	0.12, 1	0.729	758/1357 (55.9)	693/1495 (46.4)	25.71, 1	**< 0.0001**
Age group (years)								
< 5 years	103/347 (29.6)	136/403 (33.7)	1.418, 1	0.234	83/174 (47.7)	77/229 (33.6)	8.184, 1	**0.0042**
Aged 5 to 14 years	346/696 (49.7)	361/750 (48.1)	0.3603, 1	0.548	268/403 (66.5)	249/459 (54.2)	13.42, 1	**0.0002**
≥ 15 years	435/1135 (38.3)	457/1227 (37.2)	0.293, 1	0.588	407/780 (52.2)	367/807 (45.4)	7.131, 1	**0.0076**
Gender								
Male	416/921 (45.1)	420/966 (43.4)	0.5457, 1	0.46	325/545 (59.6)	314/607 (51.7)	7.262, 1	**0.007**
Female	468/1257 (37.2)	534/1414 (37.7)	0.0809, 1	0.776	433/812 (53.3)	379/888 (42.7)	19.26, 1	**< 0.0001**
Bed net use								
No	589/1413 (41.7)	638/1597 (39.95)	0.9664, 1	0.334	517/908 (56.9)	494/1058 (46.6)	20.54, 1	**< 0.0001**
Yes	296/756 (38.8)	315/ 776 (40.6)	0.5394, 1	0.463	238/444 (53.6)	197/ 434 (45.4)	5.921, 1	**0.015**

*N* total number tested, *n* number that tested positive by RDT

*P* values of less than 0.05 are significant and are in bold

Assessment of the overall diagnostic properties of both RDTs over the study period had the uRDT recording higher sensitivities than the cRDT in MTTTs 1, 2 and 4 (52.9 vs 48.8; 54.3 vs 43.8 and 59.9 vs 41.7 respectively) while cRDT had a slightly higher sensitivity only in MTTT3 (47.2 vs 44.3). The cRDT recorded higher specificities than the uRDT in all four MTTTs (90.1 vs 88.0, 92.4 vs 88.8, 94.7 vs 91.6 and 90.7 vs 84.6 respectively) (Table 5).

**Table 5:** Malaria diagnostic characteristics of RDT

MTTT	*N*		*n*		Sensitivity (%)		Specificity (%)		PPV (%)		NPV (%)	
	Kofi Kwei	Obom	Kofi Kwei	Obom	Kofi Kwei	Obom	Kofi Kwei	Obom	Kofi Kwei	Obom	Kofi Kwei	Obom
1	2178	1357	559	473	48.8 (45.5, 52.1)	53.0 (49.3, 56.4)	90.1 (88.4, 91.6)	88.0 (85.1, 90.4)	77.1 (73.4, 80.4)	84.8 (81.3, 87.7)	72.0 (69.8, 74.2)	59.6 (56.4, 62.8)
2	2131	1413	427	459	43.8 (40.2, 47.4)	54.3 (50.6, 58.0)	92.4 (90.9, 93.6)	88.8 (86.3, 90.9)	74.9 (70.6, 78.8)	82.6 (78.8, 85.8)	75.9 (73.8, 77.9)	66.6 (63.5, 69.5)
3	2208	1395	448	361	47.3 (43.8, 50.8)	44.3 (40.6, 48.1)	94.7 (93.4, 95.7)	91.6 (89.4, 93.4)	83.0 (79.3, 86.2)	83.4 (79.2, 86.9)	76.4 (74.4, 78.3)	63.4 (60.5, 66.3)
4	2380	1491	531	538	41.7 (38.6, 44.9)	59.9 (56.2, 63.5)	90.7 (89.1, 92.1)	84.6 (81.9, 86.9)	75.0 (71.1, 78.5)	77.1 (73.4, 80.5)	69.9 (67.8, 71.9)	70.8 (67.9, 73.6)

For sensitivity, specificity, PPV and NPV, the values in parentheses represent the 95% confidence intervals

*N* total number tested that had both RDT and PCR data, *n* RDT positive, *PPV* positive predictive value, *NPV* negative predictive value, *df* degree of freedom

Obom = intervention group; Kofi Kwei = control group

**Fig. 2:**
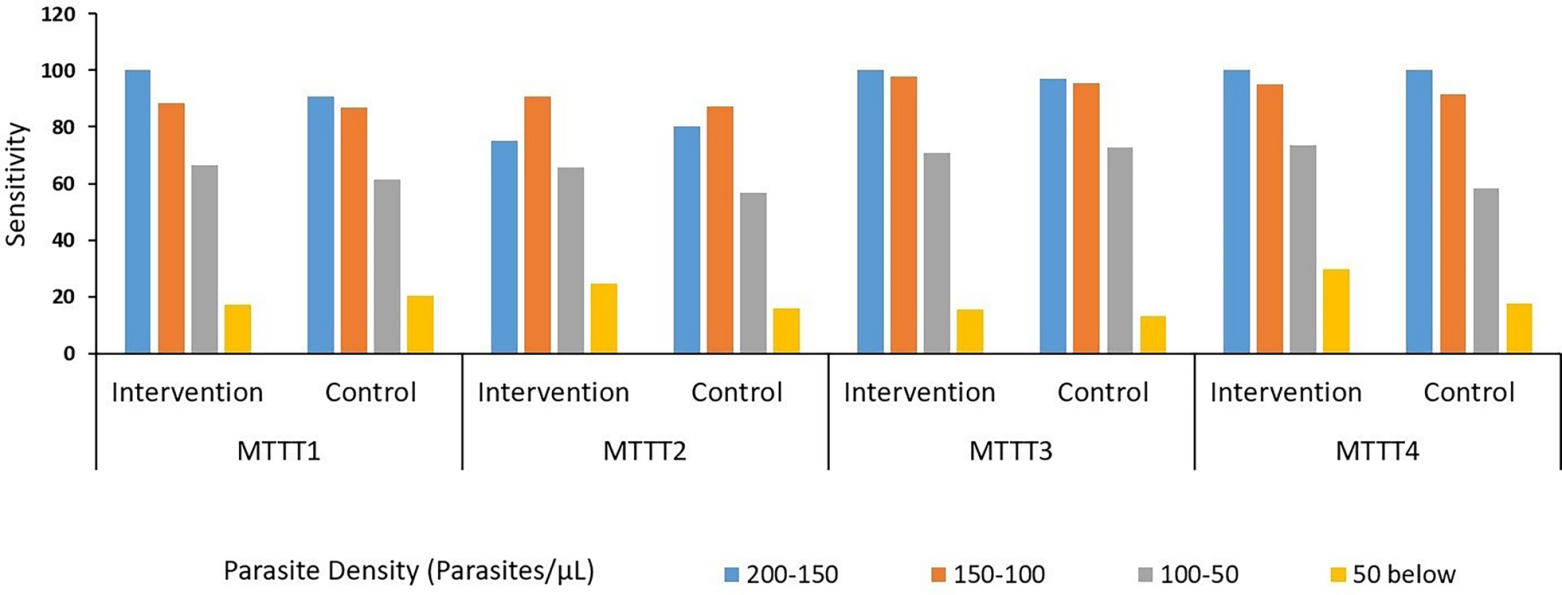
Sensitivities of RDTs used in the control and intervention arms during the study period at reducing parasite densities

When the sensitivities of RDTs were determined for participants with parasites by PCR grouped by their parasite density, both RDTs had a reduction in their sensitivities with decreasing parasite density (Fig. 2). The uRDT had a higher sensitivity than the cRDT at all of the four MTTT exercises at parasite densities ranging from 100 to 150 parasites/μl. Additionally, at parasite densities of between 150 to 200 parasites/μl, 50 to 100 parasites/μl and 50 parasites/μl or lower, the uRDT had higher sensitivity than the cRDT in three out of the four MTTTs (Fig. 2, additional file table s4).

During the home-based testing periods between the community mass test and treat interventions, the control arm recorded a higher proportion of suspected malaria cases than the intervention arm; however, the *P. falciparum* positivity rates detected by both RDT and PCR were lower than those detected in the intervention arm (Table 6). The third home-based session, which was a period over the rainy season extending from April through July to the end of September, had the highest number of febrile participants tested.

**Table 6:** Prevalence of RDT parasite positivity from the home-based testing

Test	Survey	Control, *n*/*N* (%)	Intervention, *n*/*N* (%)	*χ*^2^, *df*, *P* value
RDT	Home-based 1	24/76 (31.6)	10/24 (41.7)	0.8272, 1, 0.363
	Home-based 2	25/58 (43.1)	9/11 (81.8)	**5.5447, 1, 0.019**
	Home-based 3	77/186 (41.4)	84/123 (68.3)	**21.4598, < 0.001**
PCR	Home-based 1	18/60^a^ (30.0)	6/14^a^ (42.9)	0.8563, 1, 0.355
	Home-based 2	6/40^a^ (15.0)	5/8^a^ (62.5)	**8.5150, 1, 0.004**
	Home-based 3	70/171^a^ (40.9)	45/113^a^ (39.8)	0.0350, 1, 0.852

*N* total number of febrile individuals tested, *n* RDT or PCR positive (where appropriate), *df* degree of freedom

*P* values of less than 0.05 are significant and are in bold

Obom = intervention group; Kofi Kwei = control group

^a^DBS were not prepared for some of the samples and resulted in there being fewer samples tested by PCR than RDT

Over the course of the baseline home-based testing period (October–December 2020), there was no statistical difference in malaria prevalence in the control group and the intervention arm by RDT (31.6% and 41.7% respectively, *P* value = 0.363). However, the subsequent community cross-sectional surveys reported a higher prevalence of RDT positivity in the intervention group (Table 6).

### Modelling results

#### Estimating the effect of MTTT and mass migration with mathematical modeling

**Fig. 3:**
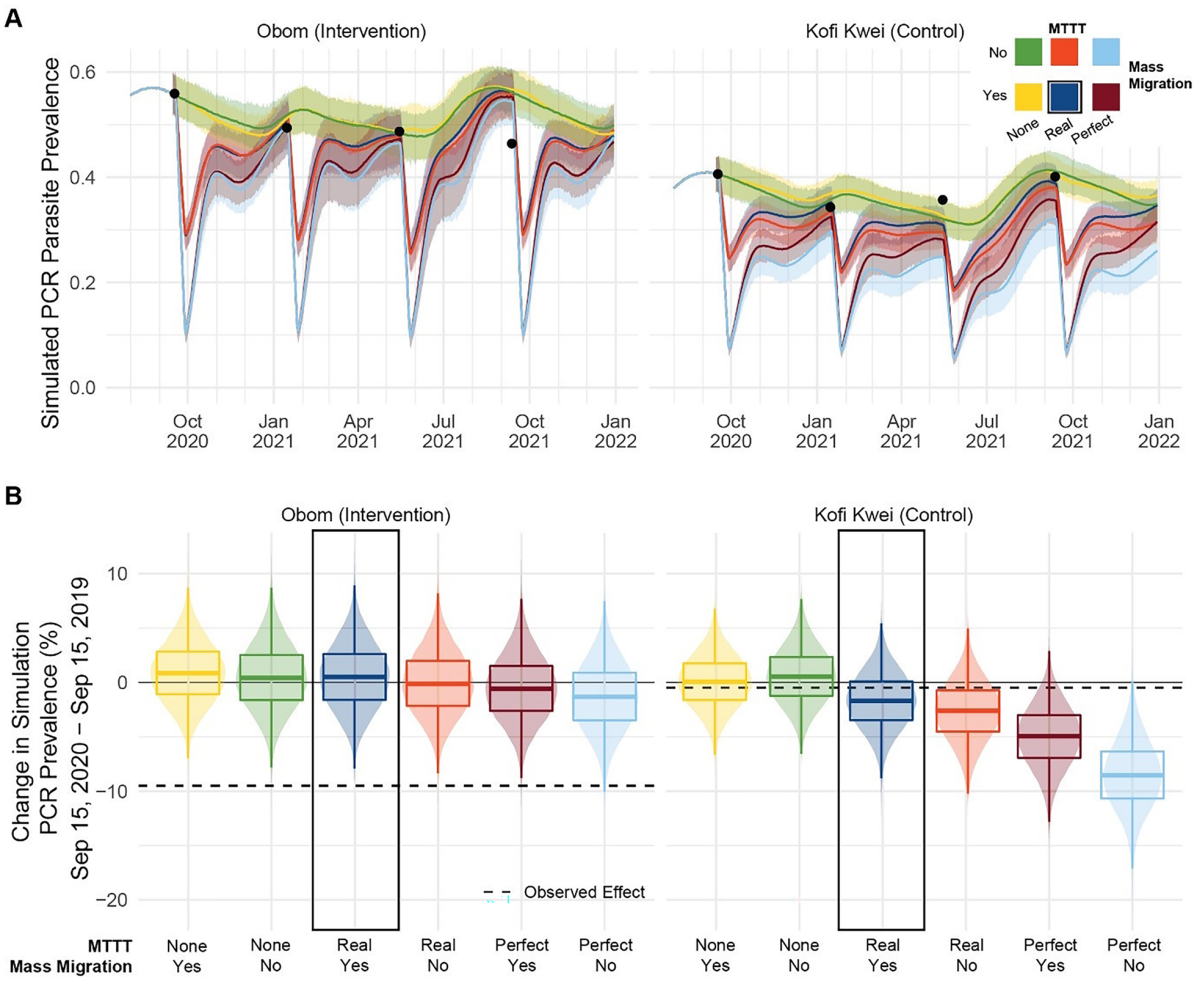
Predicted effect of MTTT interventions and mass migration on PCR parasite prevalence with a mathematical model. **A** Simulated parasite prevalence by PCR in Obom and Kofi Kwei villages from August 2020, through December 2021. Colours indicate the inclusion of mass migration events and the class of MTTT intervention in each scenario according to the legend in the upper right. Solid lines indicate the mean and shaded areas of the 95% observed intervals across 200 simulation runs. Black points indicate the PCR prevalence observed among study participants during each round of MTTT. **B** Distribution of difference in village PCR parasite prevalence from the first MTTT to the fourth by simulation scenario. Box plots indicate mean and quartiles. Dashed lines indicate the effect observed in the field

Modelled PCR parasite prevalence after 1 year of MTTT matched the observed value in Kofi Kwei and over-estimated the final prevalence observed in Obom for the scenario with MTTT and mass migration (Fig. 3A). To estimate the effect size of MTTT in the study, we compared the change in modelled PCR parasite prevalence from September 2020 to September 2021 (Fig. 3B). In Kofi Kwei, the control site, the model predicted a 1.7% decrease (95% observed interval –3.4% to 6.9%) in PCR parasite prevalence in the scenario with MTTT and mass migration, similar to the 0.5% reduction observed in the trial. For Obom, the intervention site, which had a higher starting parasite prevalence, the model predicted an absolute reduction of − 0.5% (95% observed interval − 6.4% to 5.2%) with MTTT and mass migration, whereas a reduction of 9.5% was observed in the field.

To estimate the maximal potential impact of MTTT, we used counterfactual scenarios to evaluate the change in PCR prevalence under “perfect” MTTT (using a test with 100% sensitivity and 100% specificity relative to PCR) and/or no mass migration. The simulation predicted that using a perfect diagnostic for MTTT in Kofi Kwei would result in a reduction in PCR parasite prevalence of 8.5% (95% observed interval 2.2% to 14.8%) if there had also been no mass migration. In Obom, the maximum potential impact of MTTT with a perfect diagnostic and no mass migration was 1.3% (95% observed interval − 4.9% to 7.4%). Both improving the diagnostic and removing human migration increased the effect of MTTT, particularly in Kofi Kwei.

## Discussion

The contribution of asymptomatic parasite carriage to malaria transmission has been exposed by several studies [8–10] presenting it as an important target of malaria control strategies. The assessments of impact of population-wide control strategies such as Mass Test and Treat (MTaT) and MTTT have made use of conventional RDTs which present the challenge of low sensitivity especially with parasite densities < 200 parasites/µl [3]. One of such studies estimated having missed 24.2–36.9% of infections [28]. We utilised the HRP2-based conventional RDT and Ultrasensitive RDT kits in different communities to assess the impact of MTTT on prevalence of malaria after a year. Due to the additional challenges of false-positive results associated with RDT usage, PET-PCR was used to determine the true *P. falciparum* carriage in the study populations.

Achieving a herd effect in control measures requires a high coverage of intervention. In this study, a high coverage of at least 87% of target population was achieved. In this study, research personnel were deployed with CBHVs to administer the MTTT, and the latter were also in charge of home-based testing and treatment of suspected malaria cases between mass testing and treatment intervention. This mode of administering the intervention could account for the high observed coverage as the CBHVs were instrumental in encouraging participation, reiterating the importance of the use of CBHVs in such interventions [29].

Parasite prevalence estimates were higher in Obom than in Kofi Kwei throughout the study. This suggested that the former is a higher transmission setting relative to the latter, consistent with past data on Obom as a high prevalence setting [30], which could make malaria control more challenging in Obom than in Kofi Kwei. An important finding of this study was that children aged 5 to 14 years were always the most infected during all four interventions and for all diagnostic tools. This has been observed in a number of different studies [31, 32]. Children under 5 years have been identified to be the most infected group. Although this group might be more vulnerable to complications of the disease because of limited immunity, interventions against infections seem to have been more effective in this group of children as have been observed in previous studies [31, 33, 34]. This might be because school going children often stay out longer during evenings than children under 5 years and therefore have a higher risk of infectious mosquito bites. Our observations in this study is therefore contributing to the increasing evidence that malaria epidemiology is changing with children of school-going age now being the most infected.

A major finding in our study was that the inclusion of the PCR technique in an attempt to determine the true effect of the MTTT on the parasite carriage yielded disparate results from what parasite determination using the RDTs alone produces. The PET-PCR technique yielding higher parasite prevalence than both RDTs is expected as PCR techniques have higher sensitivities than RDTs. The PET-PCR, which is also a real-time PCR technique, was reported by its inventors to have a 100% sensitivity and specificity relative to nested PCR with a limit of detection of 3.2 parasites/µl [21]. This finding suggested that assessing the impact of MTTTs by using the parasite prevalence at the start points and end points using the RDTs deployed during the exercise itself might give a false impression of the impact of the exercise. This suggests that MTTT intervention must therefore include assessment of parasite prevalence by highly sensitive molecular techniques such as PCR to assess the true parasite prevalence at the beginning and end of the MTTT.

The intervention community having a decrease in its PCR parasite prevalence at the end the three MTTTs while the control community did not record any significant reduction in parasite prevalence could be due to the high sensitivity of the uRDT plus the high (> 90%) coverage of the MTTT in the intervention arm, enabling the identification and thus treatment of more parasite reservoirs at levels that permitted herd protection against increase in parasite prevalence. The cRDT, however, with its lower sensitivity than the uRDT could not achieve this. The higher sensitivity of the uRDT than the cRDT was reiterated in the observation that the former had higher sensitivity than the latter in three out of four occasions regardless of whether the parasitaemia from all participants was considered as a whole or whether parasitaemia for individuals grouped by their parasite density was considered. Furthermore, our laboratory comparable assessment of the detection limit of the RDTs using laboratory-cultured parasites in culture medium indicated that that the uRDT had a detection limit that was fourfold lower than the lowest parasitaemia detectable by the cRDT (Additional file; table S2).

The re-establishment of parasite prevalence at the end point to levels to high (46.4%) but statistically lower than the start point level (55.9%) in the Obom, the intervention site could be attributable to a number of factors. Infected female *Anopheles* vectors hovering around during and after interventions might have played a crucial role in re-establishing asymptomatic infections after treatment and clearance of infections identified during the MTTTs [35]. Entomological inoculation rates, which could provide a fair idea of these vectors, were however not determined during these studies and vector control strategies were not directly incorporated into the MTTT exercise. These observations suggest that MTTTs should consider inclusion of vector control strategies in target communities to possibly enhance impact of MTTT interventions.

Furthermore, limited sensitivities of RDTs at low parasite densities would mean individuals carrying parasites at such low levels would be missed; thus, the true population of parasite carriers might have been missed. Such infections could maintain a supply of gametocytes at sub-microscopic densities to perpetuate infections in the community [36] and also limit what would have been a significant herd effect from treating the infected population. Mass migration could also be implicated in reducing the impact of malaria control intervention as observed in the model applied in this study. Migration could supply a population with malaria under control with new parasite species that could evade immunity developed against species that are already present in the community.

Mathematical models used to simulate counterfactual scenarios predicted that both mass migration and the limited sensitivity of the RDTs used could have diminished the effect size of MTTT in the field. These results are consistent with other studies that have investigated how human movement [37, 38] and test sensitivity [39, 40] impact the effect of mass campaigns with antimalarials, including that greater test sensitivity is needed in areas of higher transmission to adequately target the asymptomatic reservoir.

The modeled effect size of MTTT was consistent with the observed result in Kofi Kwei but not in Obom, where the model underestimated the impact of MTTT. The limited effect of the modeled MTTT in Obom may have been driven by the higher initial parasite prevalence in the village, which led to a higher modeled force of infection and thus a greater potential for resurgence after MTTT. Since even an improved test resulted in a greater predicted impact in the control village than the intervention village, it is likely that the higher baseline prevalence in Obom drove the reduced the predicted impact of MTTT in the model. Additionally, the uRDTs simulated in Obom were modeled with a higher sensitivity, but the same detection threshold, as the conventional RDTs simulated in Kofi Kwei. The ultrasensitive RDT detects PfHRP2 at lower concentrations, but this is not captured in the model. By comparing scenarios where we modeled an RDT with sensitivity and specificity of 1.0 to the trial scenario in each respective village, we saw that increasing test sensitivity and specificity alone predicted greater impact in Kofi Kwei but had no effect in Obom. This supports the idea that the added benefit of the uRDT observed in the trial is not from improved test sensitivity but from a lower detection threshold. Finally, simulations used a simplified climate model without variation in rainfall between years or between villages, and it is possible that the modeled climate is more representative of Kofi Kwei than Obom.

Limitations of this study include the fact that role of vectors in re-establishing infections after clearance was not assessed in this study. Also, the full complement of the Standard for Reporting Diagnostic Accuracy (STARD) guidelines was not used in this study by not including a diagram showing the flow of participants or using the exact order of presentation as indicated in the guidelines.

## Conclusions

At the end of the study, asymptomatic parasite prevalence at the molecular level reduced significantly in the intervention site where the uRDT was used but not in the control arm where the cRDT was used. The uRDT exhibited significantly higher sensitivity relative to the cRDT during all the surveys except for the third survey where the sensitivities of both the uRDT and the cRDT were similar. Also, highly sensitive molecular techniques such as PET-PCR should be included in parasite prevalence estimation during MTTT exercises.

### Supplementary Information

Supplementary Material 1.

## Data Availability

All data generated or analysed during this study are included in this published article.

## Abbreviations

uRDT: Ultra-sensitive NxTek eliminate RDT
cRDT: Conventional SD Bioline HRP2 RDT
PET-PCR: Photo-induced electron transfer
MTTT: Mass testing, treatment and tracking
ITNs: Insecticide-treated nets
IPTi: Intermittent preventive treatment of malaria in infants
IPTp: Intermittent preventive treatment of malaria in pregnancy
IRS: Indoor residual spraying
SMC: Seasonal malaria chemoprevention
MDA: Mass drug administration
CBHVs: Community-based health volunteers
ACT: Artemisinin-based combination therapy
DBS: Dry blood spot
CT: Cycle threshold
