# Supplementary material for: Diagnostic performance of an ultra-sensitive RDT and a conventional RDT in malaria mass testing, treatment and tracking interventions in southern Ghana
Source: Parasit Vectors. 2024 Jul 1;17:280. doi: 10.1186/s13071-024-06354-x (PMC11218287; doi:10.1186/s13071-024-06354-x)
Supplement: Supplementary file 1 — Supplementary Material 1. [file 13071_2024_6354_MOESM1_ESM.docx]

**SUPPLEMENTARY FILE**

I. Table S1: Number of study participants Present during MTTTs

| **Summary** | **Overall** | **Control Arm** | **Intervention Arm** |
| --- | --- | --- | --- |
| **Present in V1, V2, V3 & V4** | 1293 | 844 | 449 |
| **Present in V1, V2 & V3** | 1686 | 1113 | 573 |
| **Present in V1 & V2** | 2228 | 1436 | 792 |
| **Present in V1 & V3** | 1686 | 1113 | 573 |
| **Present in V1 & V4** | 1322 | 873 | 449 |
|  |  |  |  |
| **Present in V2, V3 & V4** | 1716 | 1085 | 631 |
| **Present in V2 & V4** | 1716 | 1085 | 631 |
| **Present in V3 & V4** | 2421 | 1508 | 913 |
|  |  |  |  |
| **Present in V2 & V3** | 2344 | 1463 | 881 |
| **Present in V1, V3 & V4** | 1322 | 873 | 449 |
| **Present in V1, V2 & V4** | 1322 | 873 | 449 |
|  |  |  |  |
| **Total recruited** | **Overall** | **New recruits** |  |
| **V1** | 3712 | 3712 |  |
| **V2** | 5090 | 1378 |  |
| **V3** | 5976 | 886 |  |
| **V4** | **6870** | 894 |  |

II. Detection limit of RDTs.

The SD Bioline (05CDG067B, 2023.06.02) and the NxTEK eliminated (05LDF009F, 2022.04.01) RDT kits were tested against an *in vitro* culture of *P. falciparum* parasites (NF54 isolate) according to the instructions given on their data sheets

- 14 different dilutions of an *in vitro* culture of NF54 set at 1.71% parasitaemia (determined by microscopy) and 50% hematocrit were made by diluting the starting culture two-fold using a solution containing 50% uninfected RBCs in Complete Parasite Medium.
- A total of 5 µl of sample was added to the sample well and 60 µl of buffer added to the buffer well. Result was read at 20 minutes and pictures were taken.
- The Nxtek eliminate RDT kit had a 4-fold lower detection limit compared with the SD Bioline kit

Table S2: detection limit of SD Bioline and Nxtek RDTs

| **Sample** | **SD Bioline** | **Nxtek** | **Parasitaemia (%)** |
| --- | --- | --- | --- |
| 1 | Positive | Positive | 1.71 |
| 2 | Positive | Positive | 0.855 |
| 3 | Positive | Positive | 0.4275 |
| 4 | Positive | Positive | 0.21375 |
| 5 | Positive | Positive | 0.10688 |
| 6 | Positive | Positive | 0.05344 |
| 7 | Positive | Positive | 0.02672 |
| 8 | Positive | Positive | 0.01336 |
| 9 | Positive | Positive | 0.00668 |
| 10 | Positive | Positive | 0.00334 |
| 11 | Negative | Positive | 0.00167 |
| 12 | Negative | Positive | 0.00083 |
| 13 | Negative | Negative | 0.00042 |
| 14 | Negative | Negative | 0.00021 |
| 15 | Negative | Negative | 0.0001 |

III. Health facility RDT data

We obtained monthly RDT results recorded over a 24-month period at the two study health facilities as well as two additional (Hotor and Cedikope) health facilities. The Hotor CHPS is within the Ga South municipality of the Greater Accra Region as the other two study facilities. From the chart, we notice that September of 2021 still had a high number of malaria cases relative to September of 2020 and 2019, where fewer RDT positive cases were recorded.

Figure S1: Number of RDT confirmed malaria infections in the study sites and surrounding communities

III. Supplemental Methods for mathematical modeling

*Spatial model*

All simulations were run with EMOD v2.20, a stochastic agent-based model of malaria transmission that includes human agents and a cohort model of mosquito vectors. The study villages of Obom and Kofi Kwei were modelled as separate transmission environments including site-specific rainfall and temperature data from ERA5 [1]. Geographically distinct “generic villages” were also included as nearby locations for individuals to be in when not present for the study. The GPS coordinates used to specify village locations were obtained from Google Maps and can be found, along with other village-specific parameters, in Supplementary Table 1.

**Table S3: Village-specific parameters used to construct a spatial model of the study area.**

|  | **Obom** | **Kofi Kwei** | **Obom “Generic”** | **Kofi Kwei “Generic”** |
| --- | --- | --- | --- | --- |
| Latitude | 5.735507 | 5.751757 | 5.74 | 5.74 |
| Longitude | -0.439614 | -0.405375 | -0.425 | -0.415 |
| Initial Population | 1400 | 2255 | 1475 | 1818 |
| Baseline PCR Prevalence | 0.559 | 0.406 | 0.4825 | 0.4825 |

*Vector species and biting risk*

*Anopheles gambiae s.l.* mosquito vectors were included in the model and did not move between villages. Each vector was modeled as an individual agent with temperature-dependent larval development. Within each village, vector abundance was determined by the available larval habitat, with biting risk (bites per person) distributed exponentially across the population and modulated by body surface area as a function of age [2].

*Passive case management and treatment-seeking behavior*

Simulated case management consisted of treatment with artemether-lumefantrine (AL). All treated cases were modeled as taking the full recommended course of AL with pharmacokinetics and pharmacodynamics as in [3]. Case management coverage was defined as the probability an uncomplicated or severe case would receive treatment. For management of uncomplicated clinical cases, coverage was scaled to represent age-related differences in treatment-seeking. Compared to children under 5, coverage was reduced by 20% for children 5-15 years old, and 40% for everyone age 15 or older. Severe case management coverage was set to either 0.8 or the uncomplicated case management coverage rate for children under 5, whichever was greater. Treatment was modeled with an exponential delay distribution such that individuals received treatment within 3 days for new uncomplicated cases, or 2 days for new severe cases.

*Seasonality of malaria incidence*

Seasonality of malaria in the study area was calibrated to routine reporting of all-age malaria cases at the Obom Health Center and Kofi Kwei CPHS for each month between March 2019 and September 2021. Confirmed cases were those with positive RDT result. To establish a seasonal trend, cases from both health facilities were combined for each month. The average number of monthly cases across all available years was calculated and divided by the total study area population of 4,000 to generate monthly incidence. To avoid over-fitting to abnormally low incidence reported in February, the month’s incidence value was replaced with the average of the incidence values for January and March. Seasonal trends in malaria transmission were captured in the model by scaling 1) the ratio of constant vs. temporary rainfall-driven larval habitat carrying capacities, and 2) a left-shift in the annual rainfall series, so that the shape of the simulated monthly clinical incidence resembled the smoothed monthly incidence generated from health facility data (Figure S1). An iterative calibration approach produced the following best-fit parameter values, which were used to set the seasonality in all four villages.

- Constant Carrying Capacity: **10^6^**
- Temporary Rainfall-Driven Carrying Capacity: **10^8.4^**
- Rainfall Left-Shift: **120 days**


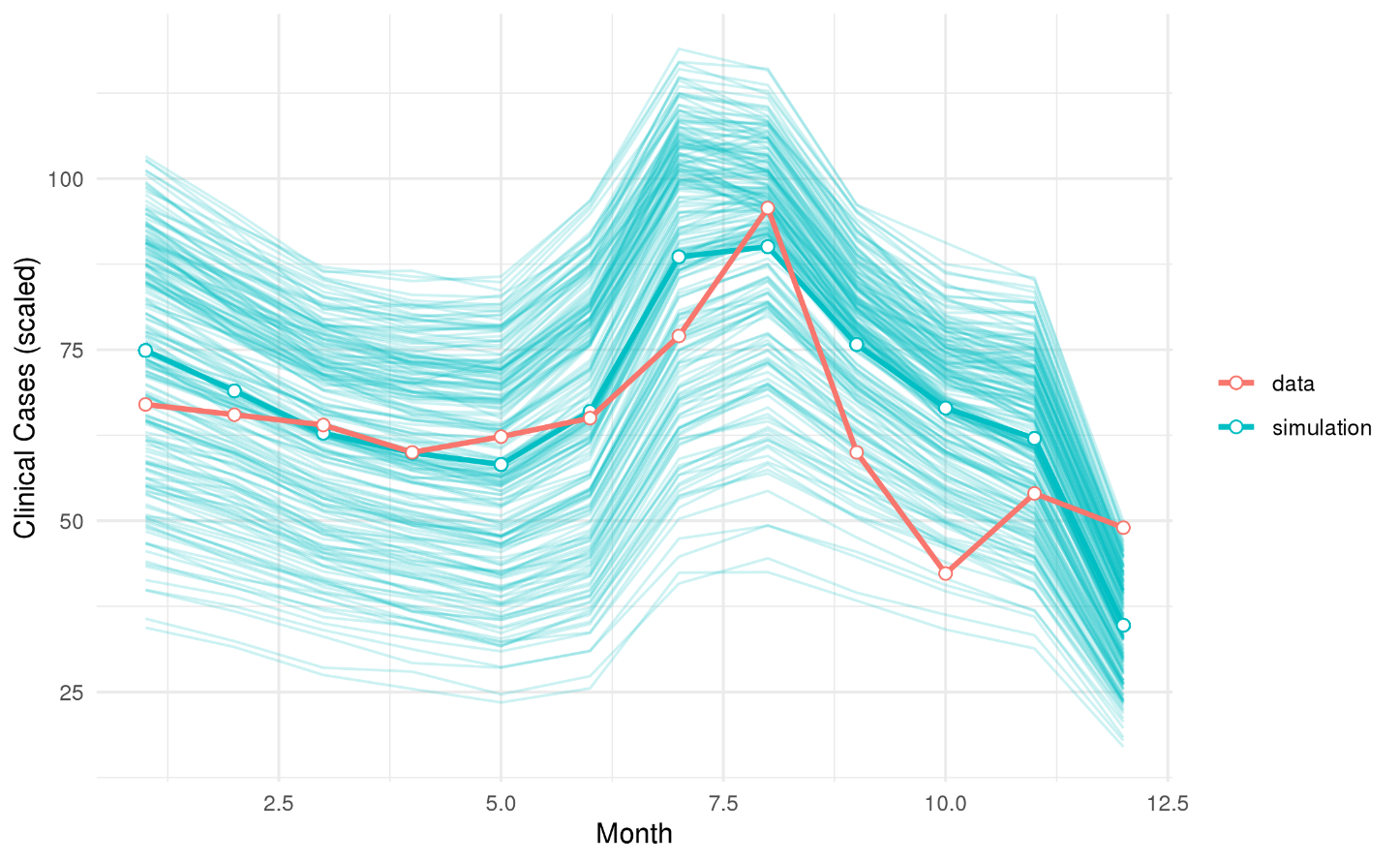


**Figure S2: Calibrating modeled rainfall and larval habitat carrying capacity to monthly incidence of clinical malaria.**  A) Monthly clinical incidence (rescaled to account for under-reporting) curve for 2,000 runs of qualitative best-fit seasonality parameter set from iterative sweeps of habitat ratios and rainfall shifts.

*Baseline transmission intensity*

Simulated baseline transmission intensity was defined as PCR parasite prevalence with a detection threshold of 0.1 parasites per microliter, at the start of mass testing on September 15^th^, 2020. Twenty larval habitat scale factors were sampled evenly in log space from 10^-0.25^ to 10^1^ for each of the villages, and an initialization phase of 30 years was run to establish population immunity in the absence of treatment. The larval habitat scaling includes the effects of historical vector control interventions like ITNs and IRS, though they are not explicitly modeled. A recurring outbreak of 1% increase in true infection prevalence each June was included to avoid permanent stochastic extinction of malaria.

Historical case management was included for a 5-year post-initialization period, corresponding to January 2016 through December 2020. Ten treatment seeking rates for children under 5 were sampled linearly from 40% (percent of children with fever who took antimalarial drugs [4,5]) to 80% (percent of children with fever for whom advice or treatment was sought [4,5]) for each village, resulting in 200 unique combinations of larval habitat and treatment seeking parameters. Treatment-seeking rates for older children and adults were as described above, scaling with treatment-seeking for children under 5.

For each combination, 10 individual stochastic realizations were performed. The simulated PCR parasite prevalence on September 15^th^ was averaged across all runs in each village under a given parameter set, compared to the initial village PCR parasite prevalence measured in the study, and ranked by increasing Euclidian distance (Figure S2). For both “generic” villages, a target prevalence of 0.4825 was chosen as an intermediate value between the measured prevalence in the two study sites.


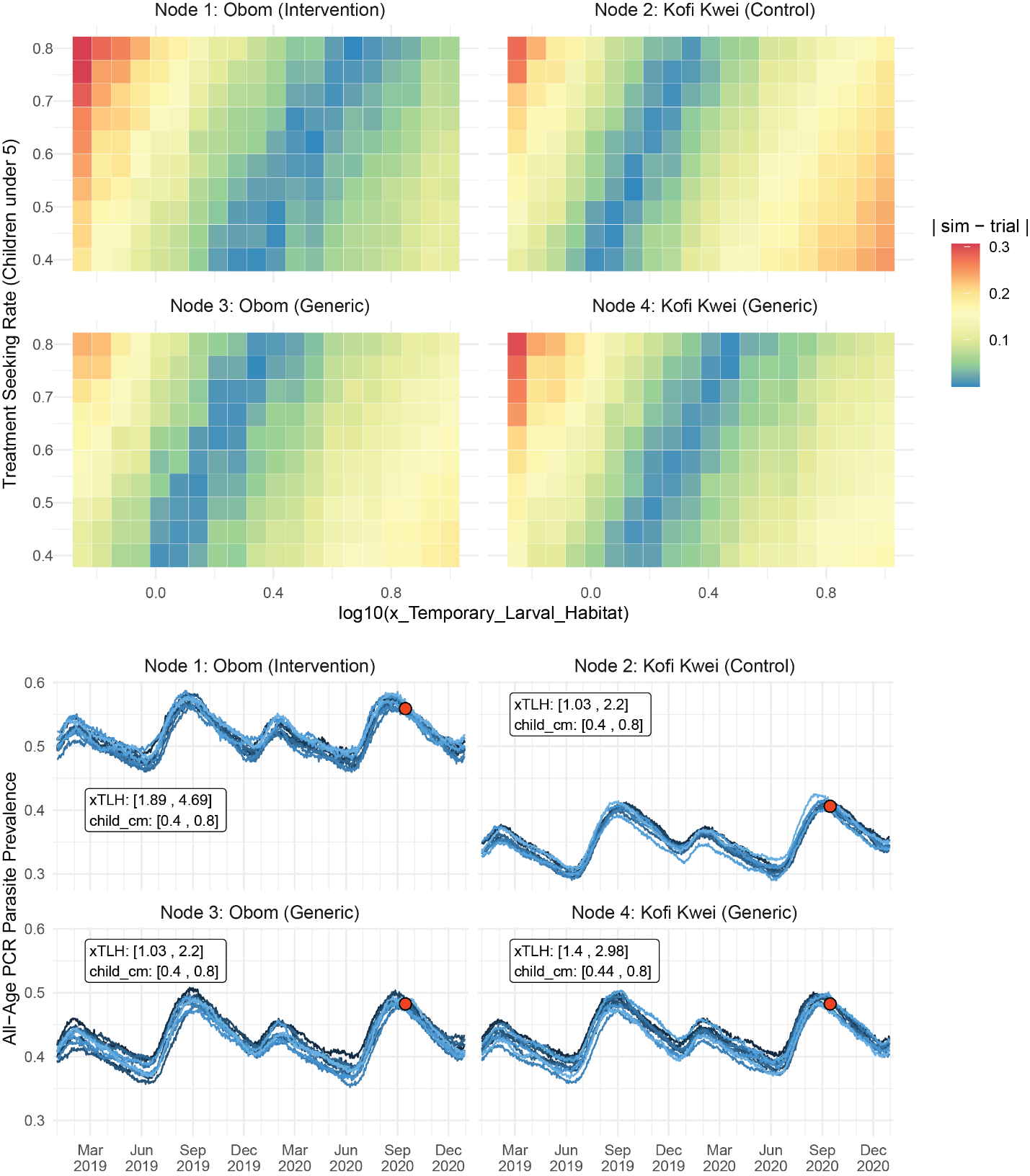


**Figure S3: Scaling larval habitats and case management coverage to target baseline malaria prevalence.**  A) Heatmap of mean ∣∣Simulated – Trial PCR Prevalence∣∣ from 10 runs of 35-year burn-in simulations across a range of habitat scales and pediatric health-seeking rates in each village. B) Timeseries of mean PCR prevalence predicted by 10 runs of simulations with the top 10 village-specific parameter combinations. Solid red points are the target village PCR prevalence at the start of the first MTTT round (September 15^th^, 2020).

*Model scenarios*

The ten top-ranked habitat scale factor and treatment seeking rate parameter sets in each node were selected and run for a 35-year initialization phase representing the years from 1985-2020, including case management in the last 5 years, to establish population immunity. The study period was then simulated over the 2-year period of January 2020 through December 2021. Our model of the study included MTTT interventions and mass migration as described above. We also modeled the following counterfactual scenarios:

- Model **with** MTTT interventions, without mass migration
- Model without MTTT interventions, **with** mass migration
- Model without MTTT intervention, without mass migration
- Model **with perfect** MTTT interventions, **with** mass migration
- Model **with perfect** MTTT interventions, without mass migration

In each scenario: 10 village-specific parameter sets x 20 stochastic realizations = 200 simulation runs. We computed the change in simulated PCR Parasite prevalence from September 15, 2020, to September 12, 2021 (dates of first and last MTTT round) and compared the means between the simulated scenarios.

*Mass testing, treatment, and tracking*

All individuals present in the study (not generic) villages underwent MTTT consisting of a simulated RDT and – if positive – treatment with AL. Tests were administered uniformly over a 10-day period during each MTTT round. The respective RDTs used in each village were modeled with sensitivities and specificities determined relative to PET-PCR results in the first MTTT round (Figure S4). The reference PET-PCR was modeled with a detection threshold of 0.1 parasites per microliter. Individuals testing positive by a modeled RDT were treated with AL.


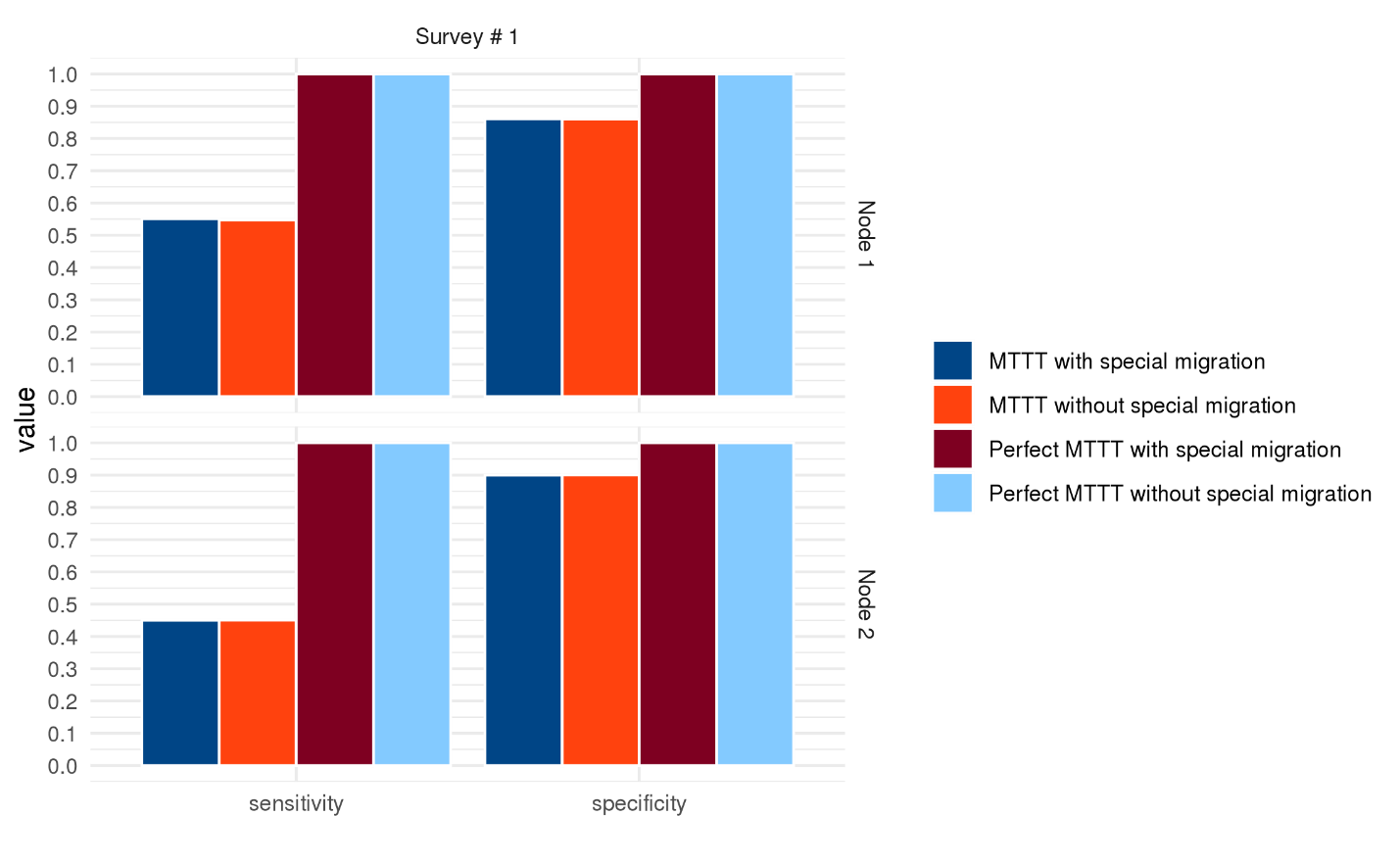


**Figure S4: Simulated test performance of modeled RDTs.** Sensitivity and specificity of the node-specific RDTs, calculated from 200 simulation runs of each scenario including MTTT. Sensitivity = True Positives Treated / All True Positives. Specificity = True Negatives Not Treated / All True Negatives. Node 1 = Obom (uRDT). Node 2 = Kofi Kwei (coRDT).

*Human movement*

All scenarios included baseline migration, where individuals moved between the study villages at a rate of 5 trips per year of 5 days’ duration each and between study and generic villages at a rate of 1 trip per year of 30 days’ duration. Additional mass-migration events were modeled in some simulations, wherein individuals were assigned migration patterns with probabilities based on participant recruitment/retention in the study. Agents representing participants enrolled after the first MTTT round began the simulation in a “generic village” and migrated to a study village before their first intervention. Missed visits were modeled as migration from the study village to its respective “generic village”. Mass migration was instantaneous with individual trips beginning anytime from 2-16 weeks after an MTTT round. The duration of these trips in and out of generic villages were set to align with the schedule of visits attended - ex. gone to generic village 120 days for 1 missed visit, 240 days for 2 missed visits, etc. (Figure S4). Vectors did not move between villages.


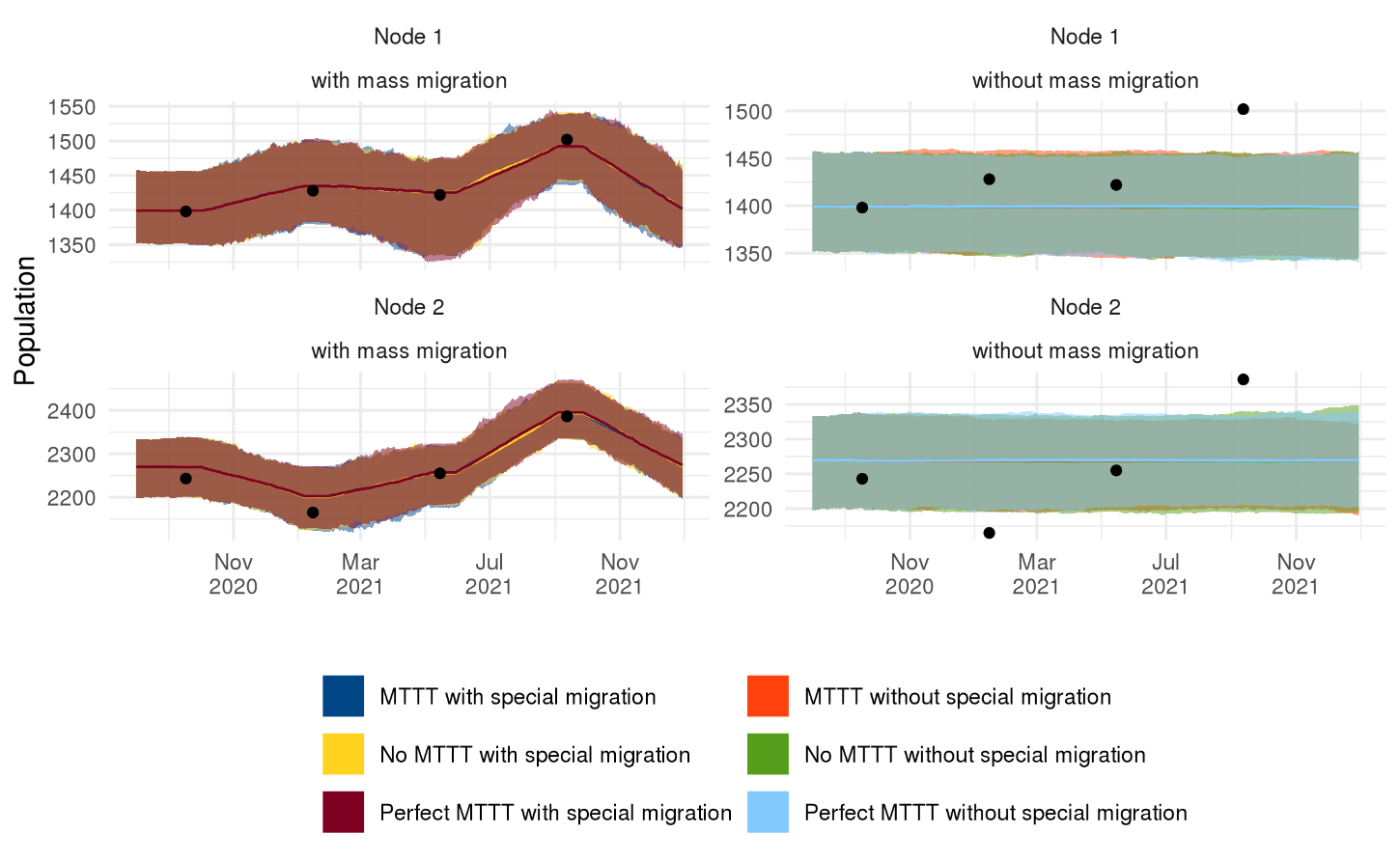


**Figure S5: Simulated mass migration to and from generic villages.** Solid lines represent the mean study population from 200 simulation runs, where shaded areas are 95% prediction intervals. Solid black points are the enrolled population enumerated at each MTTT round of the trial. Node 1 = Obom. Node 2 = Kofi Kwei.

*Additional References*

1. Chabot-Couture G, Nigmatulina K, Eckhoff P. An Environmental Data Set for Vector-Borne Disease Modeling and Epidemiology. PLoS One. 2014;9: e94741.
2. Guelbéogo WM, Gonçalves BP, Grignard L, Bradley J, Serme SS, Hellewell J, et al. Variation in natural exposure to anopheles mosquitoes and its effects on malaria transmission. Elife. 2018;7. doi:10.7554/eLife.32625
3. Gerardin J, Eckhoff P, Wenger EA. Mass campaigns with antimalarial drugs: a modelling comparison of artemether-lumefantrine and DHA-piperaquine with and without primaquine as tools for malaria control and elimination. BMC Infect Dis. 2015;15: 1–14.
4. The DHS Program Indicator Data API, The Demographic and Health Surveys (DHS) Program. ICF International. Funded by the United States Agency for International Development (USAID).
5. Ghana Statistical Service-GSS, National Malaria Control Programme-NMCP, National Public Health Reference Laboratory-NPHRL, and ICF. 2020. Ghana Malaria Indicator Survey 2019.

Table S4: Sensitivities of RDTs at decreasing parasite density during the study

|  | MTTT1 | | MTTT2 | | MTTT3 | | MTTT4 | |
| --- | --- | --- | --- | --- | --- | --- | --- | --- |
| Parasite Density | Intervention | Control | Intervention | Control | Intervention | Control | Intervention | Control |
| 200-150 | 100.00 | 90.91 | 75.00 | 80.00 | 100.00 | 97.22 | 100.00 | 100.00 |
| 150-100 | 88.28 | 86.96 | 90.77 | 87.20 | 97.70 | 95.33 | 95.04 | 91.41 |
| 100-50 | 66.35 | 61.47 | 65.58 | 56.80 | 70.75 | 72.62 | 73.64 | 58.12 |
| 50 below | 17.08 | 20.05 | 24.35 | 15.82 | 15.59 | 13.27 | 29.76 | 17.37 |
